# Seasonal occurrence of metazoan parasites in Tigerfish, *Hydrocynus vittatus* Castelnau, 1861 (Characiformes: Alestidae) from Sanyati Basin, Lake Kariba, Zimbabwe

**DOI:** 10.4102/ojvr.v86i1.1659

**Published:** 2019-08-22

**Authors:** Nyasha Mabika, Maxwell Barson, Cobus van Dyk, Annemariè Avenant-Oldewage

**Affiliations:** 1 Department of Zoology, University of Johannesburg, Johannesburg, South Africa; 2 Department of Anatomy, University of Zimbabwe, Harare, Zimbabwe; 3 Department of Biological Sciences, University of Zimbabwe, Harare, Zimbabwe

**Keywords:** *Annulotrema*, *Contracaecum*, cestode, *Lamproglena*, prevalence, aggregation

## Abstract

Lake Kariba is a tropical lake with slight variations in seasonal temperature. Temperature is an important physical variable in the biology of both fish and their parasites. Currently, there is no information on the seasonal occurrence of fish parasites in Lake Kariba. The objective of this study was to investigate the seasonal occurrence of metazoan parasites in *Hydrocynus vittatus* in Lake Kariba, Zimbabwe. Twenty fish specimens were collected by seine netting per season between October 2014 and July 2015 in the Sanyati Basin, Lake Kariba, and examined for metazoan parasites. Mean water temperatures ranged from 24.1 °C to 31.2 °C with slight variations between the seasons. Metazoan parasites consisting of Monogenea (*Annulotrema pikei, Annulotrema pseudonili, Annulotrema bracteatum*), Nematoda (*Contracaecum* larvae), Copepoda (*Lamproglena hemprichii*), Cestoda (larval cestodes, *Ichthybothrium* sp.) and Pentastomida (pentastomid larvae) were recorded. Larval cestodes were recorded in autumn and spring, while pentastome larvae were recorded in summer and spring. The *Ichthybothrium* sp. was recorded once in winter. *Annulotrema pikei* and *A. pseudonili* were observed on the gills and *A. bracteatum* on both the gills and the skin. *Contracaecum* larvae, *L. hemprichii* and *A. bracteatum* (from the skin) were recorded in all the seasons, with slight variations in prevalence, mean abundance and mean intensity. However, these variations were not statistically significant (analysis of variance or ANOVA, *p* > 0.05). The slight variations in occurrence of the parasites were probably because of the thermal stability of the lake where variation in temperature was small between seasons. Both *A. bracteatum* and *Contracaecum* larvae were aggregated on the fish host, whereas *L. hemprichii* exhibited a random distribution. Parasite diversity was at its highest during winter.

## Introduction

Fish are hosts to many protozoan, helminth and arthropod parasites (Branson & Southgate 1992). These parasites are generally in equilibrium with their hosts (Marcogliese 2005). However, there are times when changes in the environment can change the state of balance of the parasite between host and nature, thus resulting in disease (Lafferty & Kuris 1999). According to Eissa (2002), about 80% of fish diseases are because of parasite infections, especially in warm water fish; therefore, parasitic diseases remain important problems confronting modern fisheries (Ravichandran, Balasubramanin & Kannupandi 2007). Fish may also serve as paratenic or intermediate or definitive hosts of parasites, and some of these parasites can even be transferred to humans (Adams, Murrell & Cross 1997).

Some fish parasites are encountered during all seasons, some during certain seasons, or large differences occur in their seasonal abundance and prevalence. This is mainly because of physical and chemical changes in water quality, geographical isolation, inhabitancy and nourishment, or the presence and density of intermediate hosts (Möller & Anders 1986). Temperature is another important environmental variable affecting the seasonal occurrence of parasites because it affects both the host and the parasite (Xenopoulos, Lodge & Alcamow 2005).

Tigerfish, *Hydrocynus vittatus* Castlenau, 1861, are widely distributed in sub-Saharan Africa. In Southern Africa, it is the only representative of the genus *Hydrocynus* (Skelton 2001). Parasites of *H. vittatus* have been recorded in countries such as Botswana, Zimbabwe and South Africa. Most of these studies primarily focussed on single parasite groups in *H. vittatus* such as nematodes (Boomker 1994; McHugh et al. 2011), copepods (Douëllou & Erlwanger 1994), monogeneans (Christison, Van As & Basson 1998; Kunutu et al. 2018; Price, Peebles & Bamford 1969) and myxozoans (Reed, Basson & Van As 2002). To date, six *Annulotrema* (Monogenea) *s*pecies have been recorded in the African tigerfish. Paperna (1973) described *Annulotrema magna* and *Annulotrema ruahae* in Tanzania. *Annulotrema niliruahae* and *Annulotrema pikei ruahae* were also described in Tanzania by Paperna (1979). *Annulotrema pikei* was recorded in Uganda, Ghana and South Africa by Price et al. (1969) and *A. pikoides* was described in Mali (Guégan, Lambert & Birgi 1988).

Metazoan parasites consist of a big group infecting almost every organ of the fish host and may cause pathological, physiological, morphological and biochemical changes in infected tissues. Most parasites are pathogenic and weaken the host fish, thereby inducing stress in the host fish (Pardeshi, Hiware & Wangswad 2012). Parasites also affect host survival, reproduction, alter fish behaviour and migration patterns, and can even regulate fish populations and affect community structure (Garnick & Margolis 1990).

Previous studies on the parasites of *H. vittatus* in Lake Kariba have focussed on their diversity and taxonomy (Douëllou 1992; Douëllou & Erlwanger 1993; Mabika et al. 2016). These studies excluded information on the parasite community of *H. vittatus* related to spatial-temporal changes in the lake. For example, variation in the temperature has been suggested to influence the seasonal variation of parasite communities in fish (Felis & Esch 2004; Šimková et al. 2005). Studies on the seasonal occurrence of parasites may indicate periods during which epizootic outbreaks are likely to be favoured, and such knowledge is important to prevent economic losses for fisheries (Tavares-Dias et al. 2014). For this reason, the objective of the study was to investigate whether the season plays a role in metazoan parasite infections in *H. vittatus*, in the Sanyati Basin, Lake Kariba, Zimbabwe.

## Materials and methods

### Sampling of fish and examination of parasites

**FIGURE 1:**
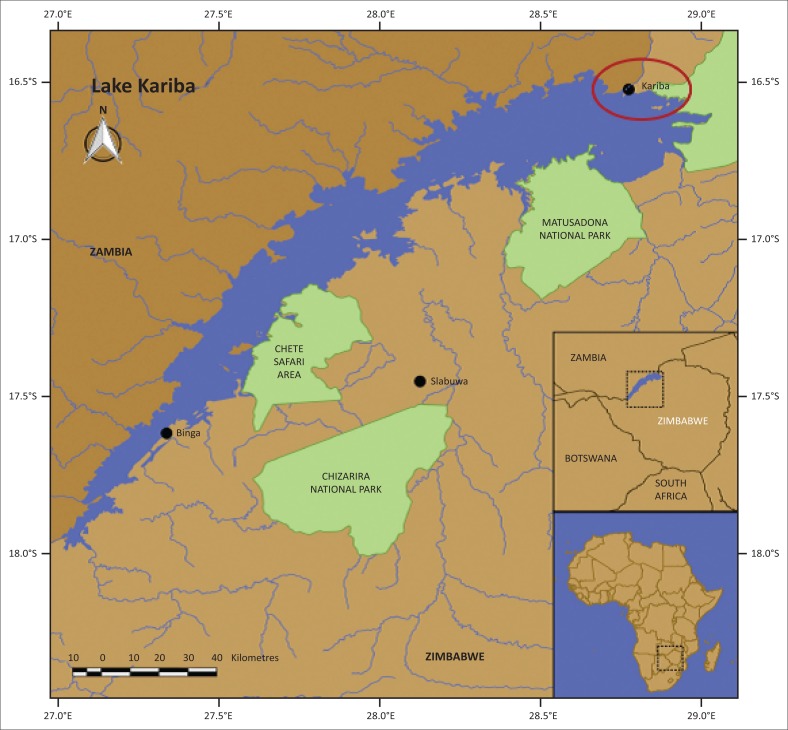
A map of Lake Kariba showing the location of the Sanyati Basin.

Sampling was carried out seasonally from October 2014 to July 2015 on the Zimbabwean side of the Sanyati Basin. Twenty fish specimens were collected during each of the four seasons using seine netting along a 15-km stretch of the northern shoreline near Kariba town (Figure 1), and also during the Kariba Invitation Tiger Fishing Tournament held at the Charara Bay in October 2014. The fish were transported live in lake water to the laboratory. In the study, the following were recorded or calculated: (1) water physico-chemical parameters, (2) infection parameters, (3) the effect of parasites on the condition factor (CF) of the host, (4) the relationship between host size and infection and (5) aggregation.

### Water quality parameters

Selected surface water quality parameters were recorded *in situ* on a seasonal basis from October 2014 to July 2015 in the Sanyati Basin, Lake Kariba. These parameters included temperature, dissolved oxygen (DO), pH and electrical conductivity. Surface water temperature and DO were measured using a HACH 330i oxygen probe, while pH and conductivity were measured using a HACH pH meter and WTW 330i conductivity meter.

Standard and total lengths (cm), weight (g) and sex (after dissection) of the fish were recorded. Parasites were collected from the fins, skin, gills, liver, body cavity, mesentery, stomach, intestines, kidney, brain cavity, swim bladder, muscle and eyes. Observed parasites were identified using the keys of Barson and Avenant-Oldewage (2006), Douëllou and Erlwanger (1994), Luus-Powell, Jooste and Junker (2008), Kuchta et al. (2012) and Kičinjaová et al. (2017). Monogeneans, *Lamproglena* sp. *Contracaecum* larvae and adult cestodes were also subjected to molecular analysis. Pentastome larvae could not be subjected to molecular analysis because of its poor fixation. Procedures for treatment, fixation, preservation and examination of parasites followed those of Madanire-Moyo and Barson (2010).

### Data analysis

The prevalence, mean abundance and mean intensity of the parasites were calculated as defined by Bush et al. (1997). The effect of the parasites on the condition of their host was determined by calculating the CF following the method of Pauly (1983):

$$\text{CF}=\text{W}\times 100/{\text{L}}^{3}$$ [Eqn 1]

where

CF is the condition factor

W is the total weight of the fish (g)

L is the standard length of the fish (cm).

Data were analysed using the Statistical Analysis System Version 9.3 (SAS 2002-2010). Normality and homoscedasticity were tested using the UNIVARIATE PLOT and Levene’s test procedures, respectively. The effect of the season on the mean abundance of specific parasite species was determined using the PROC GLM procedure of SAS in which the season was fitted as the only fixed variable in the model. Tukey’s method was used to compare the means. Regression analyses were performed to determine if fish size and fish CF were associated with the number of individual parasite species.

The magnitude of aggregation was determined following the method of Elliott (1977) by calculating the variance/mean ratio. The ratios were interpreted as follows:

- Variance and mean are approximately the same: Parasites are randomly distributed.
- Variance/mean ratio increases above 1: Parasites are aggregated.
- Variance/mean ratio is less than 1: Parasites are uniformly distributed.

### Ethical considerations

Ethical approval was granted by the University of Johannesburg Ethical Committee, Protocol January 2015 and acquisition of an appropriate research permit issued by the Zimbabwe Parks and Wildlife Management Authority (permit number 23(1)(C)(ll)09/2014).

## Results

### Water quality parameters

Mean seasonal water temperatures ranged from 24.1 °C in winter to 31.2 °C in spring (Table 1). An alkaline pH was recorded in all seasons with winter having the highest (8.2 ± 0.19) and autumn the lowest pH (7.4 ± 0.63). Mean DO was the lowest in autumn (4.7 ± 3.9 mg/L) and the highest in winter (8.5 ± 0.10 mg/L). The lowest mean conductivity (91.1 ± 15.9 *µ*S/cm) was recorded in winter. The highest mean conductivity (157 ± 100.54 *µ*S/cm) was recorded in summer.

**TABLE 1:** Value of selected physicochemical water quality parameters (mean ± standard deviation) during four seasons (October 2014–July 2015) in the Sanyati Basin, Lake Kariba.

Season	T (°C)	pH	Dissolved oxygen (mg/L)	Conductivity (*µ*S/cm)
Spring (October 2014)	31.2 ± 0.75	7.5	8.01 ± 0.56	137.5 ± 50.2
Summer (January 2015)	29.6 ± 0.51	7.8	5.46 ± 4.52	157 ± 100.54
Autumn (April 2015)	30.2 ± 0.60	7.4	4.7 ± 3.9	121.6 ± 27.68
Winter (July 2015)	24.1 ± 0.44	8.2	8.5 ± 0.10	91.1 ± 15.9

T, temperature; pH, negative of the base 10 logarithm of the concentration of hydrogen ions.

### Parasites

A total of 80 fish specimens were examined for parasites. All fish specimens were infected with at least one parasite species. Five metazoan taxa comprising monogeneans (*Annulotrema pseudonili, A. pikei, Annulotrema bracteatum*), nematodes (*Contracaecum* larvae), copepods (*Lamproglena hemprichii*), cestodes (larval cestodes, *Ichthybothrium* sp.) and pentastome larvae were recorded (Table 2). Larval cestodes were recorded in autumn and spring, while pentastome larvae were recorded in summer and spring. *Ichthybothrium* sp. was recorded once in winter. *Annulotrema pseudonili, A. pikei* and *L. hemprichii* were recorded from the gills, while *A. bracteatum* was recorded from both the gills and the skin. *Annulotrema pseudonili, A. pikei* and *A. bracteatum* could not be separated from the fish gills because they were identified from the gills by molecular analysis. The 28S and 18S rDNA (ribosomal DNA) fragments of *L. hemprichii* were distinct from other *Lamproglena* taxa. DNA analysis of *Contracaecum* larvae showed that it was closely related to the marine nematode *Contracaecum osculatum*, with a 10% difference in DNA. The DNA sequence data of the *Annulotrema* spp. and cestode were not included in this article as they will be provided in future papers focussing on taxonomy and morphology.

**TABLE 2:** Prevalence and mean intensity per season of metazoan parasites of *Hydrocynus vittatus* (*n* = 20) per season.

Parasite	Prevalence %				Mean intensity			
	Summer	Autumn	Winter	Spring	Summer	Autumn	Winter	Spring
*Annulotrema bracteatum*	30	30	45	40	24.3	19	27.11	22.4
*Contracaecum* larvae	25	20	35	25	5.25	4.9	6.3	7.8
*Lamproglena hemprichii*	20	25	40	35	2	1.6	1.7	1.9
Larval cestode	0	30	0	5	0	7	0	2
Pentastomid	5	0	0	5	1	0	0	1
*Ichthybothrium* sp.	0	0	5	0	0	0	6	0

Therefore, separating all the *Annulotrema* species would entail molecular analysis of each individual parasite and considering the abundance of these parasites, this could possibly be suggested to be performed in future studies. Consequently, their seasonal prevalence, mean intensity and mean abundance from the gills were not determined. *Contracaecum* larvae and larval cestodes were recorded from the body cavity and mesentery. The *Ichthybothrium* specimens were observed from the small intestine, while the pentastome larvae were recorded from the brain cavity and swim bladder.

### Seasonal prevalence of the parasites

*Annulotrema bracteatum, Contracaecum* larvae and *L. hemprichii* occurred throughout all seasons (Table 2). The prevalence of *A. bracteatum* was the same (30%) in summer and autumn. It reached a peak in winter and declined slightly in spring. *Contracaecum* larvae also showed slight variations in prevalence which reached a peak in winter and declined in spring. There was also a slight variation in the prevalence of *L. hemprichii* which increased from summer to winter and peaked in winter before it declined in spring. The prevalences of larval cestodes were 30% and 5% in autumn and spring, respectively. Pentastome larvae had the same prevalence (5%) in both summer and spring. The *Ichthybothrium* sp. had a prevalence of 5% in winter.

### Seasonal mean intensity of the parasites

Table 2 shows that there was a slight variation in the mean intensities of parasites throughout the seasons. *Contracaecum* larvae decreased between summer and autumn, followed by an increase in winter. Mean intensity peaked in spring, when water temperature was at a maximum. The mean intensity for *A. bracteatum* decreased from summer to autumn and reached a peak in winter when water temperature was at its lowest. The numbers declined in spring. The maximum mean intensity for *L. hemprichii* was recorded in summer. It declined in autumn and then gradually increased from winter to spring.

### Seasonal mean abundance of the parasites

Small seasonal variations in mean abundance were observed for the parasites (Table 3). Parasite numbers were lowest in autumn when DO and pH levels were also at their minimum; *A. bracteatum, Contracaecum* larvae and *L. hemprichii* decreased from summer to autumn, peaked in winter and declined in spring. Larval cestodes had a higher mean abundance in autumn than in spring. The mean abundance of pentastome larvae was the same in both summer and spring.

**TABLE 3:** Mean abundance of metazoan parasites infecting *Hydrocynus vittatus* (*n* = 20) per season.

Parasite	Summer	Autumn	Winter	Spring
*Annulotrema bracteatum*	7.05	5.28	8.2	6.24
*Contracaecum* larvae	0.41	0.38	0.55	0.49
*Lamproglena hemprichii*	0.19	0.15	0.25	0.23
Larval cestode	0.00	0.53	0.00	0.03
*Ichthyobothrium* sp.	0.00	0.00	0.30	0.00
Pentastomid	0.01	0.00	0.00	0.01

Results from the PROC GLM showed no statistically significant differences (*p* > 0.05) in the mean abundances of *A. bracteatum, L. hemprichii* and *Contracaecum* larvae throughout the seasons. Unfortunately, the sample sizes for the other parasites (larval cestodes, pentastome larvae and *Ichthybothrium* sp.) were too small to enable statistical analysis.

### Body length in relation to parasitic infection

The total number of parasites and mean intensity of infection were recorded for four different body size classes of *H. vittatus* (Table 4). The number of fish examined in each group varied and the number of parasites recovered in each respective body size group also varied. Most fish were in the 20.1 cm – 29.0 cm and 29.1 cm – 38.0 cm size classes and most parasites were recorded in the 20.1 cm – 29.0 cm group. Regression analysis showed that there was no relationship between fish size and number of parasites (*p* > 0.05).

**TABLE 4:** Total number of parasites and mean intensity in each size class of *Hydrocynus vittatus.*

Size class (cm)	*N*	Total no. of parasites	Mean intensity
11–20	5	1178	235.6
20.1–29	44	6541	148.7
29.1–38	26	5387	207.2
38.1–47	5	972	194.4

*N*, number.

### Seasonal mean condition factor of parasitised *Hydrocynus vittatus*

Seasonal variations in CFs were observed. The highest mean CFs were recorded in summer and the lowest mean CFs were observed in winter for both the male and female fish (Table 5). However, the seasonal variations in CFs were not statistically significant (*p* > 0.05). The regression analysis showed that there was no relationship between parasite abundance and CF (*p* > 0.05).

**TABLE 5:** Mean condition factor per season of parasitised female and male *Hydrocynus vittatus* per season in the Sanyati Basin, Lake Kariba.

Season	Female	Male
Summer	1.74 ± 0.15	1.65 ± 0.25
Autumn	1.60 ± 0.19	1.61 ± 0.14
Winter	1.57 ± 0.17	1.48 ± 0.33
Spring	1.58 ± 0.19	1.55 ± 0.12

### Aggregation of parasites

The data for all surveys were pooled to determine whether aggregation occurred. The variance/mean ratios of *A. bracteatum* and *Contracaecum* larvae were all greater than 1 (Table 6), indicating an aggregated distribution. However, *A. bracteatum* was more aggregated than the *Contracaecum* larvae. The variance and the mean of *L. hemprichii* were approximately the same, indicating a random distribution of the parasites on the host.

**TABLE 6:** Levels of aggregation of parasites on *Hydrocynus vittatus.*

Parasite	No. of fish hosts	Variance	Mean intensity	Variance/mean ratio
*Annulotrema bracteatum*	33	2242	29.03	77.2
*Lamproglena hemprichii*	31	1.77	2	0.89
*Contracaecum* larvae	22	34.6	4.18	8.3

## Discussion

This study for the first time investigated various aspects of the ecology of metazoan parasites in *H. vittatus* from Lake Kariba. Five metazoan parasite taxa consisting of three monogeneans, one copepod, two cestodes, larval nematodes and larval pentastomes were recorded. The parasite community of *H. vittatus* was dominated by *A. bracteatum* in all the seasons, while parasites such as larval cestodes, *Ichthybothrium* sp. and pentastome larvae were occasionally observed. The majority of the host fish were in the 20.1 cm – 29.0 cm and 29.1 cm – 38.0 cm size classes and this size class also carried the highest parasite burden. A regression analysis showed that there was no relationship between fish size and parasite infrapopulation (*p* > 0.05).

An alkaline pH was recorded for all the seasons, with the minimum recorded in autumn and the maximum in winter. Maximum pH is normally expected in autumn just after the rainy season (Buckle 1996). However, the maximum pH recorded in winter was probably because of the rains which fell late in summer and autumn. The maximum difference between the cold and warm season was 8 °C, confirming the statement by Bashirullah and Hafizuddin (2007) that temperature in the tropics varies little between seasons. Under such situations, seasonal changes in the behaviour of parasites are not expected (Bashirullah & Hafizuddin 2007). This was confirmed in the present study as there were slight variations in the occurrence of the parasites throughout the seasons. However, in temperate regions, the population dynamics of parasites such as the monogeneans is influenced by water temperature, which directly affects the parasite’s reproduction and survival (Mo 1997) as well as their existence and abundance (Koskivaara, Valtonen & Prost 1991) and determines the immune response of the host (Aaltonen, Jokinen & Valtonen 1994). While some monogeneans tend to reproduce more rapidly at a higher water temperature, others prefer cooler water temperatures (Hanzelova & Zitňan 1985). In the present study, *A. bracteatum* had the highest mean abundance in winter suggesting a preference for cooler temperatures. Minimum DO levels were recorded in autumn, while maximum levels were recorded in winter. Low levels of DO in autumn could probably be because of a reduced photosynthetic activity of aquatic plants and algae. Peak DO levels in winter could be attributed to low temperatures, increased plant and algal photosynthetic activity and strong turbulences because of the late rains in summer and autumn. Conductivity was minimum in winter, while maximum conductivity in summer could be because of a combination of anthropogenic activities and an increase in temperature. *Annulotrema bracteatum, Contracaecum* larvae and *L. hemprichii*, recorded throughout the seasons, had their maximum prevalence in winter when DO was maximum. This could possibly suggest that these parasites thrive in winter when DO is at its maximum. The other parasites (larval cestodes, pentastome larvae and *Ichthyobothrium* sp.) were not recorded throughout the seasons; hence, it was not possible to establish their prevalence in relation to DO levels.

*Contracaecum* sp. is not host-specific in the larval stage (Al-Zubaidy 2009) and the larvae usually occur in the body cavity of the fish (Whitfield & Heeg 1977). They showed a variation in prevalence and reached a peak in winter, but this was not statistically significant. According to Huizinga (1967), *Contracaecum* eggs hatch in water with an optimum temperature of 21 °C.

Though seasonal variation in the prevalence of *L. hemprichii* was observed, the variation was not statistically significant. Of note was that peak prevalence was recorded in winter, while Austin and Avenant-Oldewage (2009) recorded the maximum infection of *L. hoi* on *Labeo barbus polylepis* in Mpumalanga (temperate region) in spring and lowest in winter. This is also contrary to observations by Tsotetsi, Avenant-Oldewage and Mashego (2004) who recorded the maximum prevalence of *L. clariae* in *Clarias gariepinus* in the Vaal Dam (temperate region) during autumn and winter, with a decrease in spring and early summer.

Larval cestodes and pentastome larvae were recorded twice, while the *Ichthybothrium* sp. was recorded once. This may be attributed to ecological conditions and particularly the distribution or absence of intermediate hosts (Puinyabati et al. 2010). Single pentastome larvae were recorded in female hosts during summer and spring. According to Junker, Boomker and Booyse (1998), pentastome infections are rare in fish. Fish species that are bottom feeders are readily exposed to pentastomes while feeding on detritus or water plants (Junker et al. 1998). Top feeders such as *H. vittatus* are therefore not exposed to pentastome larvae and this could probably explain why they were rarely observed in the *H. vittatus*.

Fish of the genus *Hydrocynus* seem to be paratenic, accidental or post-cyclic hosts of both riocephalidean cestodes (Kuchta et al. 2012). This was also confirmed in this study as *Ichthybothrium* sp. was recorded only once and could possibly indicate that *H. vittatus* is an accidental or rare host.

The reproduction season for *H. vittatus* in Lake Kariba is in summer (Kenmuir 1973). According to Buchmann and Uldal (1997), it is assumed that fish are more susceptible to parasite infection during periods of reproduction. However, this is contrary to the results of the current study as parasite populations peaked in winter when the temperature was at its minimum and DO and pH were at the maximum. Minimum prevalence and mean abundance for *A. bracteatum, Contracaecum* larvae and *L. hemprichii* were recorded in autumn when DO and pH were at their lowest and the maximum in winter when pH and DO were at their peak, presumably because oxygen and pH are key factors in the population dynamics of these parasites.

There was no relationship between parasite abundance and CF and the seasonal variation in CF was also not statistically significant (*p* > 0.05). Mhlanga (2000) and Kenmuir (1973) also found no significant seasonal variation in the condition of tigerfish in the lower Sanyati River basin, although they did not investigate parasitism. However, high CFs were recorded during the breeding season, possibly as a result of developed gonads. Further research is therefore recommended to investigate whether these parasites have an effect on the condition of the fish.

The monogeneans *A. pikei, A. pseudonili* and *A. bracteatum* were all found on the gills and it was not considered feasible to separate specimens into species considering the very high number of these parasites on the gills. Only *A. bracteatum* on the skin was considered for further analysis. The variance/mean ratio of *A. bracteatum* and *Contracaecum* larvae to the number of hosts was greater than 1, indicating that the parasites were aggregated. Consequently, most fish had few parasites, particularly the monogeneans, while few fish harboured a large number of parasites. Of note was that the *A. bracteatum* were more aggregated than the *Contracaecum* larvae as evidenced by the high variance/mean ratio. However, the variance/mean ratio does not provide any information about the causes of aggregation because a number of different biological phenomena have been proposed to explain this flexible distribution (Poulin 2007).

According to Amarante et al. (2015), factors determining the degree of aggregation are not well understood. In the present study, it is assumed that the aggregative pattern could be a function of the life histories of the parasites. For example, in the case of the monogeneans which are hermaphrodites, it is assumed that they possibly aggregate after colonising their host. The aggregative distribution of monogeneans parasitising fish has also been observed by Tombi, Akoumba and Bilong Bilong (2014) in *Oreochromis niloticus*. Aggregation of the *Contracaecum* larvae could possibly be because of a single infection incident. Furthermore, *H. vittatus* is an intermediate host of the nematodes. Kennedy (1977) postulated that the aggregative distribution increases opportunities to meet a partner in order to reproduce.

The variance and mean ratio of *L. hemprichii* were approximately the same and therefore according to the approach by Elliott (1977), these parasites are not aggregated but randomly (or Poisson) distributed. Female *Lamproglena*, continuously release impregnated eggs into egg sacks, from which larvae hatch continuously, scatter and thereafter each complete an intricate free living life cycle before the impregnated parasite female becomes infective to another host (Madanire-Moyo & Avenant-Oldewage 2013), hence the random infection pattern.

## Conclusion

The slight seasonal variations in the occurrence of the parasites could probably be because of the thermal stability of the lake. *Annulotrema bracteatum* and *Contracaecum* larvae were aggregated on the fish host, whereas *L. hemprichii* exhibited a random distribution. Parasite diversity was the highest in winter and parasite abundance did not correlate with the season.
